# On the Treatment of Scarlatina

**Published:** 1811-04

**Authors:** 


*To the Editors of the Medical and Physical Journal.*

GENTLEMEN,

As it is hardly possible to receive too much evidence in support of any eligible practice that is not generally adopted, I venture to send the following remarks for insertion in your valuable Journal.

Since the beginning of the last spring I have spent some time with two extensive and worthy medical practitioners in Suffolk and Norfolk, and I have had an opportunity of witnessing their method of treating Scarlatina Anginosa, a disease which for several months nearly superseded every other. A plan calculated to counteract every inflammatory symptom was invariably recommended and persevered in; repeatedly purging with calomel, &c. spunging the body with a cold fluid, and in the warm weather freely exposing it to the air, low regimen, scarification of the fauces, and, in short, all the antiphlogistic remedies enumerated by Mr. Hamilton

Hamilton (page 102) except phlebotomy*. This was performed only in a few cases (adults) none of which terminated fatally. Effervescing draughts with carbonate of soda and tartaric acid † were constantly given. This treatment was succeeded by the most happy result; and whenever any occasion occurred of comparing its effects, in similar circumstances, with those of an opposite treatment, where wine and nourishing diet were allowed from the commencement, its superiority was frequently evinced by the absence of danger and a more speedy recovery.

That so varying a disease as Scarlatina, which sometimes assumes the most formidable appearances, should be wholly disarmed by the most simple means, is not to be expected. Many have died, under my observation, of the remote consequences of the disease, two or three weeks, or even months from its first attacking them, of abscess, dropsy, mortification, consumption, &c. I have, however, neither seen nor been informed of a single instance, where the antiphlogistic system had been embraced early and adhered to strictly, that ended mortally in a few days; although I have known many patients, in the same neighbourhood, and almost on the same spot, who, after every stimulating tonic and antiseptic means

* In 1788, the scarlet fever was unusually prevalent in some part of Norfolk. An experienced medical man, who was in the midst of it, sent me the subsequent account which I relate in his own words. "Insatiable thirst at the beginning of the disease, pulse very quick, soreness of the throat so as to prevent deglutition, delirium after a few days, and then scarlet eruption. They who had the eruption earlier became easier, and mended sooner, and their pulse were lessened to eighty, yet the tongue and fauces were still lined with a matter of a yellow appearance. The *vis vitæ* shewed great signs of assisting itself by the eruption, by diarrhœa and profuse sweatings, and the younger subjects were always relieved by these. The aged mostly sunk under the disease, their pulse becoming thready and not above thirty. In all their speech was remarkably affected; they could scarcely articulate a word. Bleeding at the nose gave great relief, and those who applied early were blooded and emetic tartar given, until the stomach was well emptied. As soon as the inflammatory symptoms decreased we began with antiseptics, ordering port wine, bark, and a liquid diet consisting of strong broths." He adds, "*All those who underwent blood-letting in the beginning of the disease recovered.*"

† The tartaric acid is perhaps as good as the citric in its effects, and to be preferred on account of being less expensive. Every person who is at all acquainted with chemistry, knows why that acid should be mixed with carbonate of soda rather than of potass: a small quantity only of the compound formed by the latter mixture being soluble in water, chief of it subsides to the bottom.

 had had been tried, died in the manner related by Mr. Goodwin, on the 5th or 6th day, or sooner. The only case that I have seen of this kind, was a healthy strong man, above thirty years of age, who called in no medical assistance until the third day, when he was in a hopeless and most dreadful state, low delirium, pulse scarcely perceptible, a glossy eye, tongue brown and hard, inability of articulation, skin of a dry burning heat, and all over of a dark copper colour, the whole body furnishing a most noxious fœtor. His throat could not be examined: he died in six or eight hours. This poor man's symptoms for the first thirty hours had, as far as I can learn, nothing in them of peculiar malignancy. After walking a few miles and drinking a little, he was taken in the evening with a cold chill and head-ache; was afterwards very hot; throat stiff, not very sore; and he thought himself but slightly unwell all the next day. I know not why we may not be allowed an opinion, that the active and timely exhibition of proper remedies would have saved him from so quick and shocking an end.

It would greatly assist us in estimating the value of Mr. Hamilton's paper on Scarlatina, to be informed whether he has met with any fatal instances from the immediate effects of the disease, or whether the cases he has given us should be regarded as comprising a summary of his whole experience on that head, and as warranting a conclusion, that his practice has been attended with universal success.

I believe there are not a great many people, at the present time, who do not think the antiphlogistic plan of treatment as successful, in most cases of Scarlatina Anginosa, as what is called the stimulating and antiseptic; and I am convinced there are none who, after a fair trial, will hesitate to pronounce it, in the first stage of every case, far preferable. If such a trial should, in the least degree, be furthered by the recommendation here given, it will be highly gratifying to the wishes of, Gentlemen,

Your most humble and obedient Servant,

SENEX.

*February* 12, 1811.

